# Enhanced Optical Management in Organic Solar Cells by Virtue of Square-Lattice Triple Core-Shell Nanostructures

**DOI:** 10.3390/mi14081574

**Published:** 2023-08-09

**Authors:** Pavithra Gattu Subramanyam, Narayan Krishnaswamy, Koushik Guha, Jacopo Iannacci, Eze Nicholas Ude, Venkatesha Muniswamy

**Affiliations:** 1 Department of Electronics & Communication Engineering, Sai Vidya Institute of Technology, Bangalore 560064, Karnataka, India; pavi.svit@gmail.com (P.G.S.); venkatesha.m@saividya.ac.in (V.M.); 2 Department of Electrical & Electronics Engineering Sciences, Visvesvaraya Technological University, Belagavi 590018, Karnataka, India; 3 Department of Artificial Intelligence & Machine Learning, NITTE Meenakshi Institute of Technology, Bangalore 560064, Karnataka, India; narayank101@gmail.com; 4 Department of Electronics and Communication Engineering, National Institute of Technology, Silchar 788118, Assam, India; koushik@ece.nits.ac.in; 5 Center for Sensors and Devices (SD), Fondazione Bruno Kessler (FBK), 38123 Trento, Italy; 6 Computer and Robotics Education, University of Nigeria, Nsukka 410105, Nigeria; nicholas.eze@unn.edu.ng

**Keywords:** triple core-shell nanoparticles, plasmonic effect, organic solar cell, absorbance, FDTD method

## Abstract

This research focuses on enhancing the optical efficacy of organic photovoltaic cells, specifically their optical absorbance and electrical parameters. The absorbance of photons in organic solar cells (OSCs) was studied by incorporating an optical space layer and triple core-shell square-lattice nanostructures. For better chemical and thermal stability, a dielectric-metal-dielectric nanoparticle can be replaced for embedded metallic nanoparticles in the absorption layer. The 3D (finite-difference time-domain) FDTD method was used to analyze the absorption and field distribution in OSCs using 3D model morphology. Firstly, an optimization of thickness of the optical spacer layer was analyzed and secondly, the impact of adding triple core-shell nanostructures at different levels of an OSC were studied. The photovoltaic properties such as short circuit current density, power conversion efficiency, fill factor, V_oc_ were investigated. The proposed design has demonstrated an improvement of up to 80% in the absorption of light radiation in the photoactive region (donor or acceptor) of OSCs in the wavelength range of 400 nm to 900 nm when compared with that of nanostructures proposed at various layers of OSC.

## 1. Introduction

Solar energy harvesting through photovoltaic technologies is an emerging field of study among all other renewable energy sources due to its advantages, which include flexibility in mechanical design, low cost, and a low-temperature production process [1,2]. Also, over the recent years, OSCs have demonstrated an improvement in power conversion efficiency [3]. Ongoing research efforts in OSCs aim to address their limitations and improve their efficiency. Researchers are exploring various approaches, including the development of new materials, interfaces, device structures, and optimization techniques, to improve the performance of OSCs and bridge the efficiency gap with perovskite solar cells. The study of OSC has potential uses for energy harvesting within, in wearable technology devices [4], in biomedical technology [5], indoor organic photovoltaics [6], green house applications [7], etc. The efficiency of OPV cells has increased as a consequence of the identification of novel conjugate polymers and small-molecule elements. Using these novel materials, researchers have been able to achieve efficiency of up to 4%. 

P3HT-Poly(3-hexylthiophene) is a standard donor material for soluble organic semiconducting polymers and is therefore widely used in organic electronics research. In terms of light-harvesting characteristics, PCBM ([6,6]-Phenyl C61 butyric acid methyl ester) is a commonly used acceptor material [8]. Numerous researchers have taken a keen interest in thin-film OSCs.

In order to make OSCs more useful for photovoltaics, a lot of research has been done to improve the light trapping in the active region [9,10,11,12,13]. The stability and diminished effectiveness of OSCs are the primary obstacles [14]. The improvement of light absorption in OSCs is being studied using a variety of light-trapping techniques, some of which involve the introduction of the surface plasmon effect, which is caused by metallic nanostructures, which also includes hybrid nanostructures [15,16,17,18], photonic crystals [19,20], microcavities, and the sandwiching of a spacer layer (SL) of material zinc oxide (ZnO) or titanium suboxide (TiOx) between the active region and the metal electrode [21,22]. The latest research trend is focusing towards perovskite solar cells. The role of plasmonic light trapping in reducing optical losses in perovskite solar cells is still not fully understood. Further research is needed to investigate the extent to which plasmonic nanoparticles (NPs) contribute to increased absorption and their potential for enhancing the electrical properties of both OSCs and perovskite solar cells [23].

Metallic nanostructures permit surface plasma excitation, which is used to enhance the performance of nanoparticles in long-wavelength regions. Due to their unique optical properties, metal nanoparticles can be used as scattering centers in solar cells. The optical spectra of metallic nanoparticles are distinguished by the presence of a visible or near-infrared resonance frequency band. The surface plasmon resonance band (SPR) indicates the presence of localized surface plasmons in metal nanoparticles. On prefabricated solar cells, metallic nanoparticles can be separated from the semiconductor’s surface by using a dielectric material layer that functions as a passivation layer. Localized surface plasmon resonance (LSPR) excites nanoparticles in response to incoming sunlight, which then illuminates the optically denser environs of the solar cell [24].

LSPR excitation enhances the ambient optical fields of metal nanoparticles. Photons entering the metal nanoparticles are either absorbed or scattered by the heat transfer into the surrounding space. If the environment is highly absorbed or captivated, a portion of the intensified light fields will travel through it and be absorbed by it, which, in the worst-case scenario, could be distributed as heat within the metallic nanoparticles. However, nanoparticles still exhibit undesirable absorption. In solar cells, however, preferential scattering on metal nanoparticles is advantageous, as light scattered within the medium is collected and converted into electricity. Typically, as the size of metallic nanoparticles increases, more light can be scattered as opposed to absorbed.

Recent research investigations demonstrate that metal nanoparticle absorption can be prevented by incorporating dielectric-metal core-shell nanoparticles [25,26]. The preservation of solar cells and nanoparticles has received relatively little attention in the literature, despite the fact that there have been several attempts to improve OSC performance by capturing light in the organic layer. By adding a silica coating on silver nanospheres (Ag@SiO_2_ NSs) to the OSC, the lifespan of the solar cell is increased. Additionally, it enhances the light-trapping properties of dark and bright fields. By substituting ZnO for SiO_2_, the intrinsic light absorption of a P3HT:PCBM photoactive layer was enhanced [2]. The optical behavior of gold (Au) clad with silica (core-shell) nanospheres (Au@SiO_2_ NSs) inserted into different thicknesses of PEDOT: PSS deposit was also studied [18]. To enhance the efficacy of plasmonic inverted OSCs, the same research team proposed an alternative method involving an ultrathin SiO_2_ layer coated with silver nanoparticles (Ag@SiO_2_). Their primary drawbacks are their short longevity due to oxidation and their high production cost due to the incorporation of gold or silver nanoparticles.

The presence of a SiO_2_ shell prevents the direct interaction of excitons (quasi-particles) with metal nanoparticles, thereby minimizing excitons’ recombination at the surface of metallic nanoparticles. Furthermore, the presence of a SiO_2_ shell limits light scattering from nanoparticles’ surfaces [27]. Consequently, receiving additional benefits from the LSPR effect in enhancing light energy absorption is hindered. By introducing a novel triple core-shell spherical nanoparticle structure as SiO2@Ag@SiO_2_, the local field enhancement factor and light scattering cross-section were enhanced, thereby increasing power absorption. There has been research on providing details on synthesis of triple core-shell nanostructures [28,29,30]. In this study, we sought to enhance light energy absorption and trapping in the active (P3HT:PCBM-donor/acceptor) layer. 

## 2. Mathematical Modelling 

It is important to design, simulate, and analyze the optical properties of an organic photovoltaic cell before fabricating the device. For complex geometries, the 3D FDTD is a modernized means for solving Maxwell’s curl equations in nonmagnetic materials that employs Maxwell’s equation [31]:

(1) $$\nabla \times E=-\mu \;\frac{\partial H}{\partial t}$$

(2) $$\nabla \times H=\varepsilon \;\frac{\partial E}{\partial t}$$

Faraday’s law is represented by Equation (1), and Ampere’s law is given by Equation (2), where *E*, *H*, *ε*, and *μ* are the electric field, magnetic field, the permittivity, and the permeability of the material, respectively. FDTD is also a powerful tool for analysis of different structures of polymer solar cells.

The simulation space in the FDTD method is divided into similar cubic cells. The way every component of a magnetic field is bounded by components of an electric field determines the positions of electric and magnetic fields’ component in each and every cell, and is similar the other way vice versa.

Maxwell’s equations can be discretized, and new six mathematical expressions are obtained for six components of electric and magnetic fields by application of the finite-difference method in spatial and time components. The vertices of a 3D non-uniform lattice are represented by 1D coordinates:

(3) $$\left(X_{p}{,\;p=1,\;N}_{p}\right);$$

(4) $$\left(Y_{q}{,\;q=1,\;N}_{p}\right);$$

(5) $$\left(Z_{r,\;}{\;r=1,\;N}_{r}\right);$$

The identical cubic cells obtained by dividing the simulation space, which is a non-uniform space; the cell and edges in this non-uniform space are given by:

(6) $$\left\{{\;X}_{p+\frac{1}{2}}{=X}_{p}+\left(\frac{∆X_{p}}{2}\right)\right\};$$

(7) $$\left\{{\;Y}_{q+\frac{1}{2}}{=Y}_{q\;}+\left(∆\frac{Y_{q}}{2}\right)\right\};$$

(8) $$\left\{{\;Z}_{r+\frac{1}{2}}{=Z}_{r\;}+\left(∆\frac{Z_{r}}{2}\right)\right\};$$

The electric and magnetic field in the non-uniform grid is given by equations:

(9) $$E_{X}{|}_{p+\frac{1}{2},\;q,\;r}^{n}\equiv {\;E}_{X\;}{(X}_{p+\frac{1}{2}}{,\;Y}_{q}{,\;Z}_{r},\;n∆T)$$

(10) $$H_{X}{|}_{p,\;q+\frac{1}{2},\;r+\frac{1}{2}}^{n+\frac{1}{2}}\;\equiv {\;H}_{X}{[X}_{p}{,Y}_{q+\frac{1}{2}}{,\;Z}_{r+\frac{1}{2}},\left(n+\frac{1}{2}\right)∆T]$$

The surface integrals and contour integral are applied over the lattice cells’ face and over the boundaries of the bounding face. Equations (9) and (10) are obtained by using discrete field approximations over cell faces.

Evaluation of time derivatives by using difference approximations results in

(11) $${E\;}_{X}{|}_{p+\frac{1}{2},\;q+1,\;r\;}^{n}(∆X_{p})\;-{\;E}_{X}{|}_{p+\frac{\;1}{2},\;q,\;r}^{n}\;(∆X_{p})\;-{\;E}_{Y}{|}_{p+1,\;q+\frac{1}{2},\;r}^{n}\;(∆Y_{q}{)+E}_{Y}{|}_{p,\;q+\frac{1}{2},\;r}^{n}\;(∆Y_{q})=\;-\left[\mu_{p+\frac{1}{\;2\;},\;q+\frac{1}{2},\;r}\left(\frac{H_{Z}{|}_{\left(p+\frac{1}{2},\;q+1,r\right)}^{\left(n+\frac{1}{2}\right)}-H_{Z}{|}_{p+\frac{1}{2},\;q+\frac{1}{2},\;r}^{n-\frac{1}{2}}}{∆\;T}\right)+M{|}_{p+\frac{1}{2},\;q+\frac{1}{2},\;r}^{n+\frac{1}{2}}\right]∆X_{p}∆Y_{q}$$

(12) $$H_{X}{|}_{p,q+\frac{1}{2},r+\frac{1}{2}}^{n+\frac{1}{2}}h_{X_{p}}-{\;H}_{X}{|}_{p,q-\frac{1}{2},r+\frac{1}{2}\;}^{n+\frac{1}{2}}h_{X_{p}}-H_{j}{|}_{p+\frac{1}{2},q,r+\frac{1}{2}\;}^{n+\frac{1}{2}}h_{Y_{q}}{+H}_{Y}{|}_{p-\frac{1}{2},\;q,\;r+\frac{1}{2}}^{n+\frac{1}{2}}h_{Y_{q}}=\;-\left[\varepsilon_{p,\;q,\;r+\frac{1}{2}}\left(\frac{E_{Z}{|}_{p,\;q,\;r+\frac{1}{2}}^{n+1}-{\;E}_{Z}{|}_{p,\;q,\;r+\frac{1}{2}}^{n}}{∆T}\right)+\frac{\sigma_{p,\;q,\;r+\frac{1}{2}}}{2}\left(\frac{E_{Z}{|}_{p,\;q,\;r+\frac{1}{2}}^{n+1}{+E}_{Z}{|}_{p,q,\;r+\frac{1}{2}}^{n}}{∆T}\right){+J|}_{p,\;q,\;r+\frac{1}{2}}^{n+1}\right]h_{X_{p}}{\;h}_{Y_{q}}$$

where $h_{X_{p}}=(∆X_{p}+∆X_{p-1})/2;\;$*p*= 2, $N_{x}$ and the $h_{Y_{q}}=(∆Y_{q}+∆Y_{q-1})/2;\;$*q*= 2, $N_{Y}$, $\varepsilon_{p,q,r+\frac{1}{2}}$ is averaged permittivity, $\sigma_{p,q,r+\frac{1}{2}}$ is averaged conductivity, and $\mu_{p+\frac{1}{2},\;q+\frac{1}{2},r}$ is averaged permeability about grid edges.

The law of conservation of energy is used to calculate the absorption coefficient, or the absorption spectra *A_s_ (λ)* can be obtained by:

(13) $$A_{s}\left(\lambda \right)=1\;-Y$$

(14) $${Y=R}_{s}\left(\lambda \right)-{\;T}_{s}\left(\lambda \right)$$

where λ represents operating wavelength, $R_{s}$ is reflectance, and $T_{s}$ is transmittance.

Organic solar cells have the thickness of a photoactive layer that can be equivalent to the sunlight wavelength, causing standing wave effects that may change the possibility of optical absorption. At any point *x* in the photoactive layer, the absorbed optical power density can be stated as [32]:

(15) $$Q\left(x\right)=\frac{1}{2}{\;c\varepsilon }_{0}\alpha \eta |E\left(x\right){|}^{2}\;$$

where *c* is the speed of light, $\varepsilon_{0}$ permittivity in vaccum, η is the real part refractive index, and *E(x)* is the electric field at *x* position. By transfer matrix formalism, the field *E(x)* may be determined. By measuring transmission and reflection using UV–Vis spectroscopy or an ellipsometer, one may determine the total amount of absorption in a film. The film thickness variation of the different material was chosen to optimize optical absorption. Optical properties such as transmittance, reflectance, and absorbance can be obtained by considering the different values for thickness of material and the optical constant of materials used in an organic photovoltaic cell.

**Figure 1:**
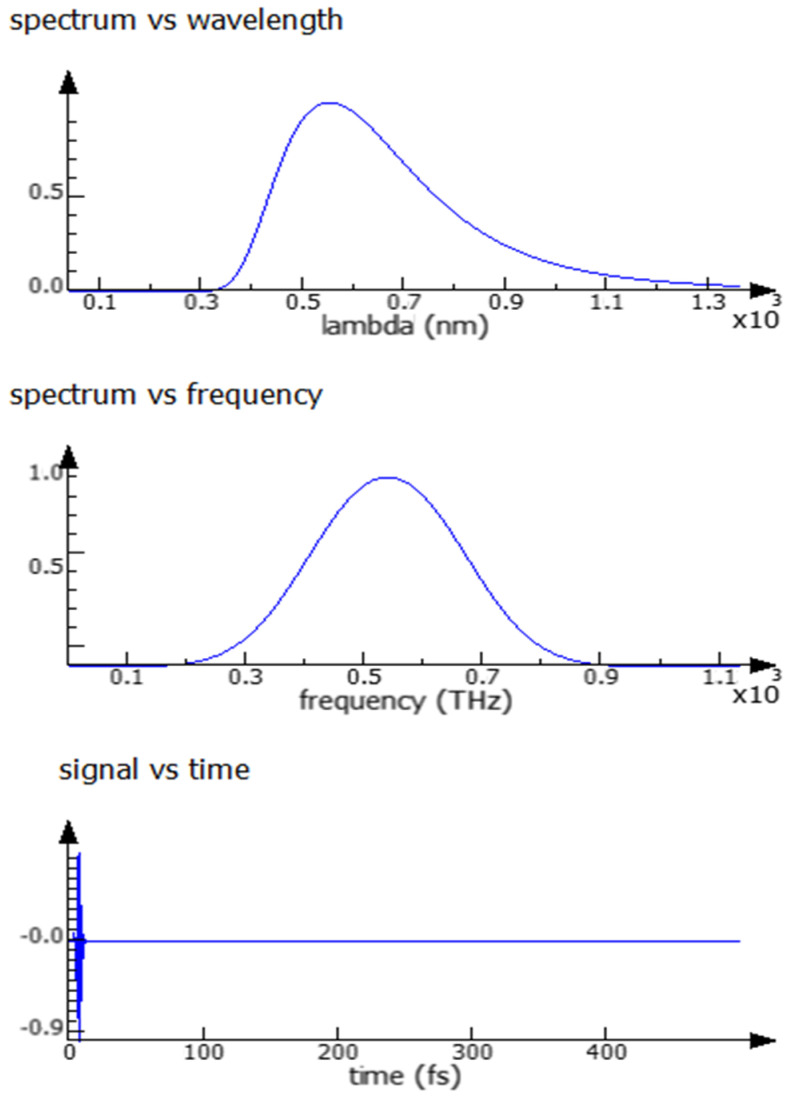
Plane wave source in OSC simulation.

The frequency band of solar radiation can be calculated using the simulation tool for optical analysis based on the 3D-FDTD method. Spectra computed from the FDTD source are automatically introduced from Gaussian pulses, as shown in Figure 1 below.

The light incident on the OSC can be considered according to the AM 1.5 solar power spectrum. The generation rate in the photoactive layer is considered as every electron-hole pair generated by each absorbed photon. The plane wave source is used as an incident light source at normal incidence in the z-axis direction between the wavelength of 400 nm and 900 nm. The finest mesh size for the simulation is selected at 1 nm. Boundary conditions chosen for the simulation setup are periodic set along both x and y directions; a perfectly matched layer (PML) at the z direction at the glass substrate and at the aluminum layer. A PML was chosen to avoid parasitic reflections in the z direction.

**Figure 2:**
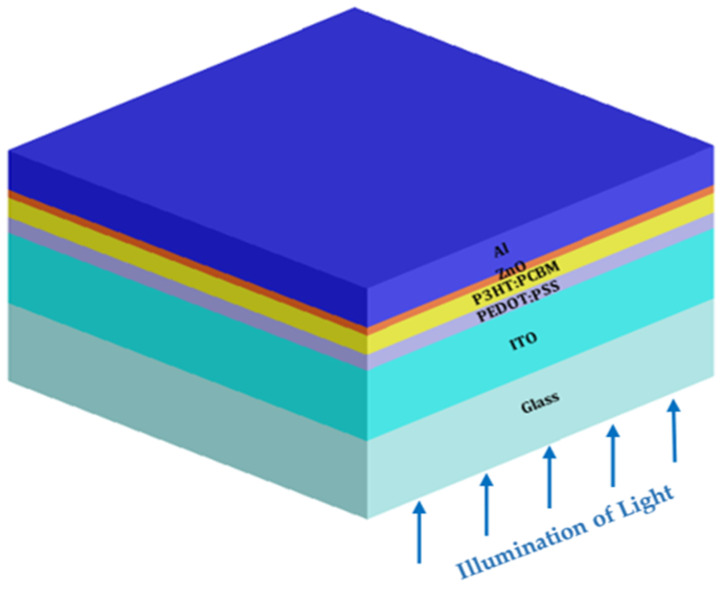
Prospective view of a standard organic solar cell (OSC) considering a ZnO-buffer layer.

In conventional architecture of OSCs shown in Figure 2, indium tin oxide (ITO) acts as the anode, and its work function is between the highest occupied molecular orbital level (HOMO) and the lowest unoccupied molecular orbital level (LUMO). PEDOT:PSS is a hole transport layer (HTL) that has several uses on the basis of its excellent transparency in the visible light range, near IR (700 nm to 1 mm wavelength), and near UV (100 nm to 400 nm wavelength). In polymer-small molecule architecture, P3HT is frequently utilized as a donor polymer, while PCBM is employed as an acceptor polymer for small molecules. In such devices, PCBM acts as a wide-bandgap acceptor and improves absorption in the short wavelength area, while P3HT acts as a low-bandgap donor and helps to increase absorption in the long wavelength region. Due of its ease of applications and excellent solubility in chloroform, chlorobenzene, dichlorobenzene, and toluene—common solvents for P3HT—PCBM is favored over other small molecules. Due to their stability, high electrical conductivity, high electron affinity, and outstanding electron mobility, ZnO materials, one of the group II-VI binary compound semiconductors, have been taken into consideration for solar cell applications.

**Figure 3:**
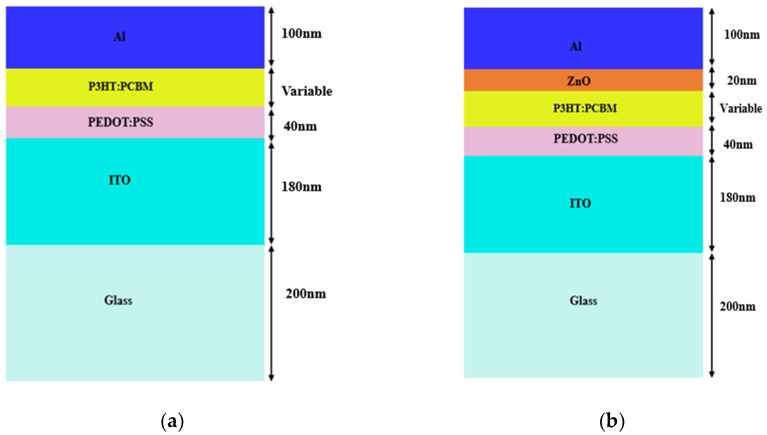
Schematic of the standard structure of OSCs (**a**) without a ZnO optical spacer and (**b**) with a ZnO optical spacer.

The proposed design has been modeled and simulated using 3D-FDTD software. The thickness of the layers used in the model was as depicted in Figure 3. The thicknesses were fixed according to the literature at: Al—100 nm, ZnO—20 nm, P3HT:PCBM—varied from 30 nm to 50 nm, PEDOT:PSS—40 nm, ITO—180 nm, Glass-SiO_2_—200 nm [2]. As we know that organic materials often exhibit low absorption, particularly within a narrow range in the visible spectrum and due to its lower mobility, a thickness increase rises the internal series resistance and, thereby, reduces the fill factor. To increase the power conversion efficiency (PCE) of organic PV cells, methods for light trapping can be utilized to enhance light absorption inside the cells’ thin active layers. Since the internal quantum efficiency (IQE) of OPV cells tends to decrease as the active layer thickness increases, light trapping is also crucial for enhancing OPV cell performance. Thin active layers are preferred in order to achieve a high IQE while retaining the benefits of newer light trapping techniques [33]. 

**Figure 4:**
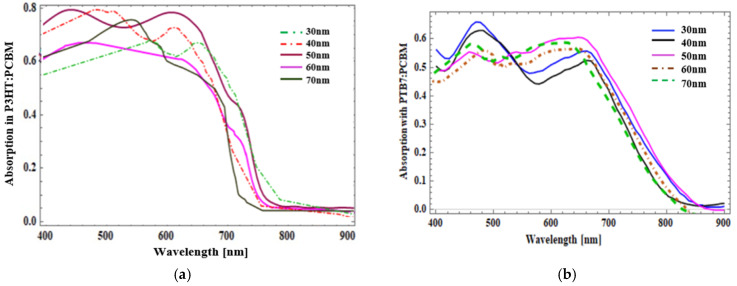
Absorption spectra in the active layer for different thicknesses varying from 30 nm to 50 nm: (**a**) P3HT:PCBM; (**b**) PTB7:PCBM.

The design was simulated for various active layer P3HT:PCBM thicknesses and PTB7:PCBM thicknesses ranging from 30 nm to 70 nm. The plots in Figure 4 show the wavelength versus absorption spectra across wavelength ranges of 400 nm to 900 nm. From Figure 4a,b, the P3HT:PCBM active layer thickness of 50 nm has the best photon absorption, hence, this material and thickness was chosen for further investigation.

**Figure 5:**
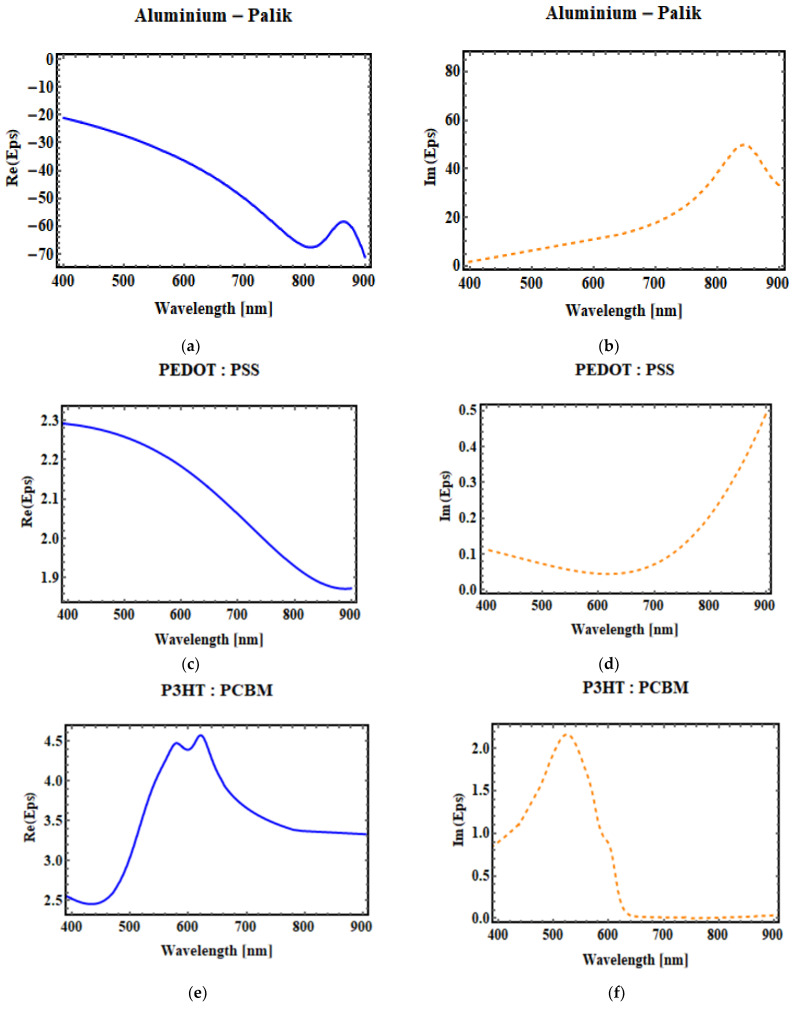

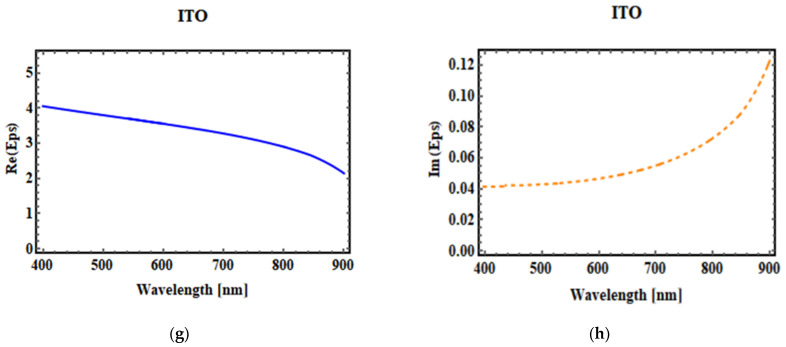
Real part (**a**,**c**,**e**,**g**) of aluminum permittivity, PEDOT:PSS permittivity, P3HT:PCBM permittivity, and ITO permittivity. Imaginary part (**b**,**d**,**f**,**h**) of aluminum permittivity, PEDOT:PSS permittivity, P3HT:PCBM permittivity, and ITO permittivity, which is based on the Lorentz–Drude mode and operates in the 400–900 nm wavelength range.

Table 1 shows the absorption wavelength of the photoactive material which acts as a donor and acceptor in the OSC along with the orbital energy levels. The real and imaginary parts of the permittivity of aluminum (Al), P3HT:PCBM, PEDOT:PSS, ITO are shown in Figure 5. The FDTD solver database was used to determine spectral dependencies of optical characteristics of the materials used, such as the refractive index and absorption coefficient.

## 3. Simulation Analysis of Proposed Nanostructures in Organic Solar Cell

### 3.1. Conventional OSC without Optical Spacer Layer

**Figure 6:**
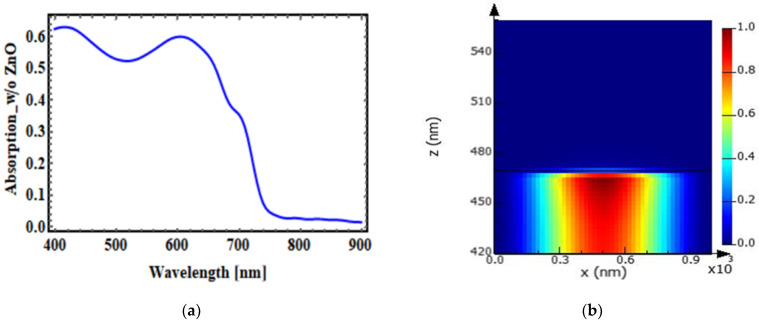
(**a**) Absorption in the photoactive layer without ZnO in a conventional OSC. (**b**) Mode profile.

In this section, we analyzed the conventional OSC without the optical spacer layer and have shown the absorption plot as shown in Figure 6a; the modal analysis has been plotted in Figure 6b. It shows the absorption in the photoactive layer without an optical spacer layer, that is, without a ZnO layer, and it was up to 60–65% in the wavelength range between 400 nm and 700 nm. Absorption decreased with rising wavelength. Figure 6b shows the mode field profile analysis of a conventional OSC without an optical spacer layer.

### 3.2. Conventional OSC with Optical Spacer Layer

**Figure 7:**
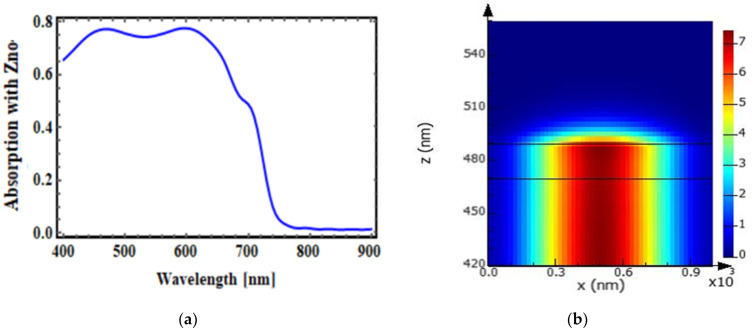
(**a**) Absorption in the photoactive layer with ZnO in a conventional OSC. (**b**) Mode profile. The black lines indicate the structure of OSC is superimposed on to the image of figure showing mode profile.

This section represents the absorption plot of a conventional OSC that included a zinc oxide optical spacer region. The absorption plot in Figure 7a clearly shows that the absorption in the photoactive (donor-acceptor) layer with the ZnO optical spacer region was around 80% in the wavelength range between 400 nm to 700 nm. Figure 7b shows the mode field profile analysis conventional OSC structure with an optical spacer layer, and it is apparent that the absorption of light was increased.

The optimization of ZnO (zinc oxide) thickness in the OSC structure can play a significant role in enhancing light absorption and redistributing the optical electric field. It also plays an essential role in the device as it extracts electrons from the layer of photoactive material. This prevents recombination that occurs when charge carriers migrate towards their respective electrodes. In this paper, we look at how the thickness of the ETL-ZnO affects solar cell absorbance. Strong electron transport characteristics and transparency to solar radiation when light is received via it are the key features of ETL. As a result, between the active layer and the electrode, ZnO was chosen as the electron transport layer. The optical properties of ZnO, such as its refractive index and absorption characteristics, can beneficially redistribute the optical electric field within the OSC structure. The presence of a ZnO layer alters the optical field distribution, influencing how light interacts with the active layer. This redistribution can enhance the interaction between the incident light and the photoactive materials, improving light absorption and charge generation [34]. ZnO materials are broad bandgap semiconductors having a UV-only absorption band gap of 3.1–3.3 eV [35]. When a ZnO layer is introduced, it can aid in preserving the active layer by lowering the number of interfacial defects, which is useful for trap-free bimolecular recombination at a constant rate. Additionally, ZnO can be linked with substances with lower energy gaps, which includes dye sensitizers, polymers made of organic materials, and semiconductors with reduced band gaps, to increase its ability to absorb light in the visible spectrum.

**Figure 8:**
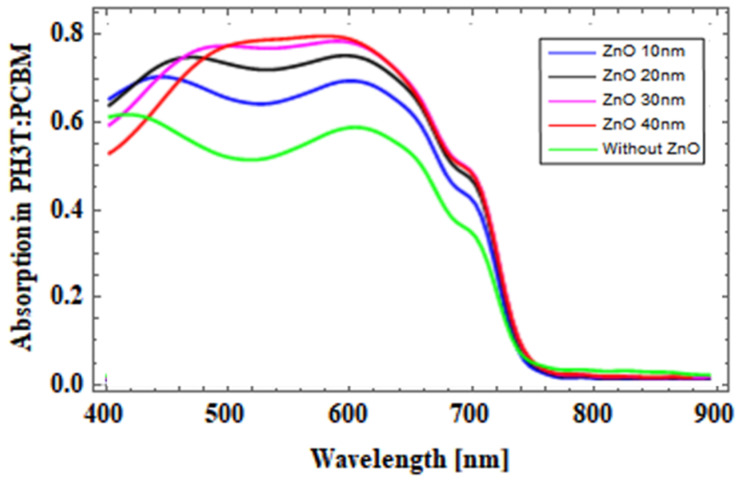
Absorption in active layer in a conventional OSC with variation in thickness of the ZnO layer.

The effect of variation in the thickness measure of the ZnO region in the OSC was analyzed, and absorption in the photoactive layer is plotted in the graph presented in Figure 8. The ZnO layer thickness was varied from 10 nm to 40 nm with a step size of 10 nm. From the absorption plot it can be observed that the absorbance of light in the active region also varied with changes in the thickness measure of the ZnO layer.

It is evident from the simulation results that the absorption was at a higher percentage when compared to that of the conventional OSC without a ZnO optical spacer layer. For improvement of light absorption, it is important to optimize ZnO thickness, which helps to boost the electromagnetic field distribution within the P3HT:PCBM photoactive layer.

### 3.3. Design for Organic Solar Cells with Proposed Triple Core_Shell Nanostructures

Traditionally, the maximum absorbance in metals and suitably n-doped semiconductors NPs, LSPR frequency approximation in a spherical nanoparticle or a spherical shape can be predicted by the Drude model [36] as given in Equation (16) below:

(16) $$\omega_{\mathrm{LSPR}\;}=\left(\frac{\omega_{p}{}^{2}}{\varepsilon_{0}+2\varepsilon }\right){\;}^{\frac{1}{2}}$$

where $\varepsilon_{0}\;$ is the dielectric constant, and ε is dielectric function. By ignoring the shift in the imaginary axis and considering the behavior of inner electrons, the above relation can be a derivative from the surface plasmon resonance state, and permittivity of bulk material can be related. When a surface plasmon is localized in a nanoparticle of a size comparable to or smaller than the light wavelength used to stimulate the plasmon, a localized surface plasmon (LSP) is produced.

The following Equation (17) defines the plasmon’s frequency:

(17) $$\omega_{p}^{2}=\frac{{4\pi N}_{e}}{m_{e}^{e_{f}}}$$

where *N_e_* is the free-electron density and $m_{e}^{e_{f}}$ is the electron’s effective mass [37].

**Figure 9:**
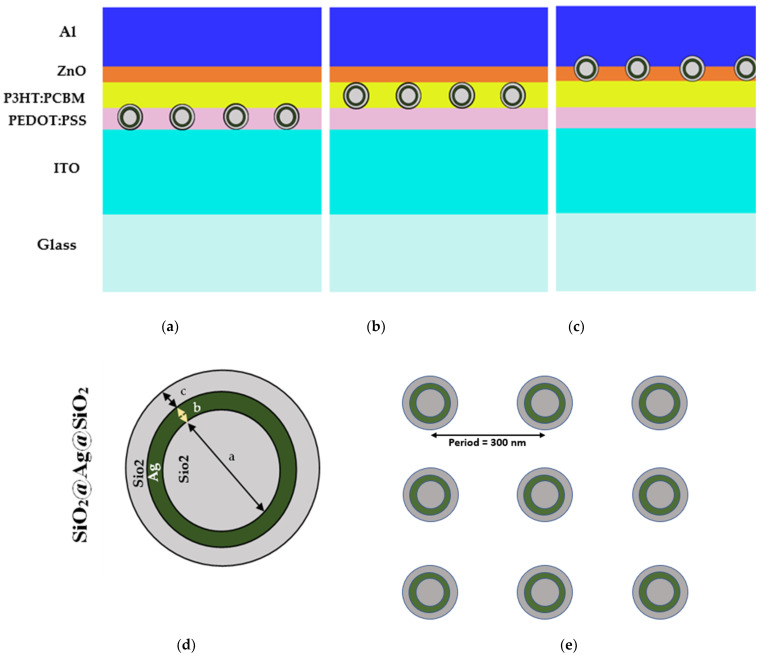
Schematic structure of proposed method embedding SiO_2_@Ag@SiO_2_ nanospheres (**a**) above the ITO layer; (**b**) above the PEDOT:PSS layer; and (**c**) above the P3HT:PCBM layer. (**d**) Dimensions of triple core-shell nanospheres. (**e**) Square lattice structure with period of 300 nm.

In this work, square-lattice triple core-shell nanospheres were introduced with a period of 300 nm at various layers of organic solar cells for optical analysis. In the category of metal-dielectric nanoparticles (NPs), the metal component occupies a significant portion of the NPs and can lead to optical losses. Typically, there exists a direct correlation between the size of the metal and the optical losses in NPs. However, this correlation becomes less significant or can be disregarded in the case of very small nanoparticles. Research on metallic nanoshell materials, such as Au and Ag, has demonstrated that silver nanoshells exhibit higher optical efficiency and superior plasmonic properties. This advantage can be attributed to the reduced optical losses compared to those of gold. Additionally, investigations into core and capping layer materials, such as SiO_2_ and TiO_2_, have revealed that SiO_2_ has minimal influence on the plasmonic properties of nanoparticles. This is due to the close proximity of the real part of SiO_2_’s dielectric function to that of Ag. Furthermore, SiO_2_ coating not only inhibits exciton recombination on the Ag surface but also enhances the chemical and thermal stability of the plasmonic nanostructure. Figure 9 shows the schematic of introducing SiO_2_@Ag@SiO_2_ nanospheres above the ITO layer, above the PEDOT:PSS layer, and above the P3HT:PCBM photoactive layer, as represented in Figure 9a–c. The color variation in the Figure 9a–c represents different layers of proposed organic solar cells as mentioned in Figure 3b.

The dimensions of the triple core nanosphere shown in Figure 9d are defined as a = 30 nm SiO_2_ core, b = 1 nm thickness of Ag layer, c = 1 nm thickness of SiO_2_ shell. Due to LSPR effects in triple core-shell nanospheres, there was an enhancement in light trapping and the absorption of power. The scattering from the surface (outer layer) of the metal nanoparticles (NPs) was limited by the contact of excitons with the surface of metal NPs and also limits its recombination, hence, a SiO_2_ layer was introduced.

Table 2 shows the summary of the short circuit current density Jsc (mA/cm^2^), the generation rate (1/m^3^/s), and other performance parameters for the different OSC structure designs as specified. It was observed that the short circuit current density was enhanced by the inclusion of an optical spacer and plasmonic effects. This improvement may be attributable to the SiO_2_@Ag@SiO_2_ NSs that were embedded in the active layer as well as the inclusion of a ZnO optical spacer layer, both of which induced an increase in scattering and absorption. In conventional OSCs, the position of the NSs above the PEDOT:PSS boosts light harvesting within the active layer by an increased concentration of the electromagnetic field surrounding the SiO2@Ag@SiO_2_ NSs coupled with plasmon resonance. The improved short circuit current density helped increase the fill factor and was also compared with the short circuit current density in an OSC structure without NSs, which was 9.50935 mA/cm^2^ in this work, and was reported as 10.631 mA/cm^2^ in [38]. From the table below, we can note that J_sc_ was increased to 14.0162 mA/cm^2^, which, in turn, helped obtain increased power conversion efficiency of 10.02%.

**Figure 10:**
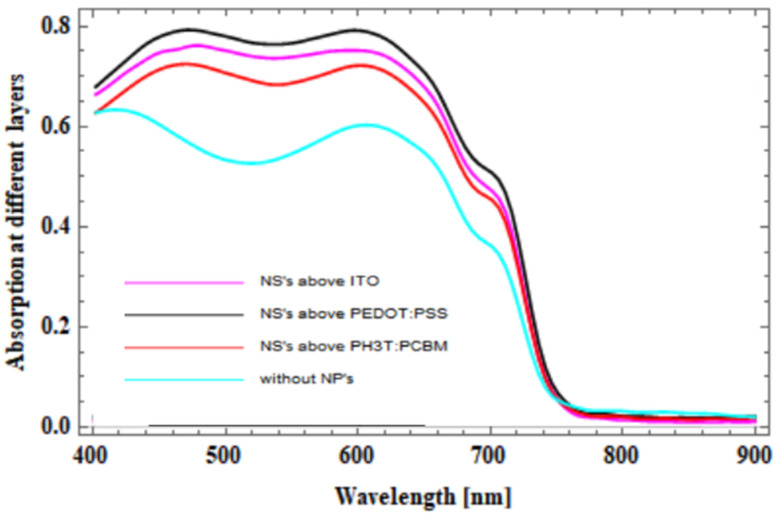
Absorption in the active region with and without triple core-shell SiO_2_@Ag@SiO_2_ nanospheres.

The absorption of light at different layers of an OSC with the introduction of square-lattice SiO_2_@Ag@SiO_2_ triple core-shell nanospheres was plotted in the graph shown in Figure 10. Light trapping in the photoactive layer was the compared with the light absorption in the work proposed in [23,39,40]. We can say that the effect of triple core-shell nanospheres above the PEDOT:PSS layer was at a higher percentage of 80%, compared to introduction of triple core-shell nanospheres in the other layers of OSC with a ZnO optical spacer layer. The different methods and techniques for fabrication of core-shell nanoparticles have been investigated in recent years [41], which helps select the appropriate method for suitable applications.

## 4. Conclusions

Analyses were conducted on the enhancement of light absorption by square-lattice SiO_2_@Ag@SiO_2_ triple core-shell nanospheres in three distinct regions or layers of the OSC. To investigate the enhancement of light absorption, FDTD and CHARGE simulation modules were used. The simulation results indicate that SiO_2_@Ag@SiO_2_ triple core-shell nanospheres could substantially improve the total absorption of organic photovoltaics with respect to their optical response. Due to the effect of local field enhancement and the scattering effect of the proposed nanospheres, the absorption in the active region was increased by up to 80% between the wavelength range of 450 nm and 650 nm for the SiO_2_@Ag@SiO_2_ triple core-shell nanospheres introduced above the PEDOT:PSS layer. Optimal absorption was also observed when the triple core-shell nanostructures were incorporated above the PEDOT:PSS layer with efficiency of 10.02%. This research aids in further analysis of the properties of organic photovoltaics and their various applications.

## Figures and Tables

**Table 1:** Wavelength of absorption (in nm) for photoactive acceptor material and donor material.

Sl.No	Material of Photoactive Region	Wavelength (nm) of Absorption	Orbital Energy Level
1	P3HT	300–550	LUMO: −3 eV HOMO: −5 eV
2	PCBM	200–700	HOMO: −6.5 eV LUMO: −4.3 eV

**Table 2:** Performance parameters of the OSC structure design.

OSC Structure Design	Current from Simulation Volume	Short Circuit Current Density J_sc_ (mA/cm^2^)	Max Generation Rate (1/m^3^/s)	V_oc_ (v)	FF (%)	PCE (%)
Without ZnO layer	3.02177 × 10^−11^ A	7.58529 mA/cm^2^	1.5266 × 1028 1/m^3^/s	0.60	53.0	2.41
With ZnO layer and without NSs	3.78948 × 10^−11^ A	9.50935 mA/cm^2^	1.5515 × 1028 1/m^3^/s	0.64	64.85	3.94
Triple core NSs above ITO	3.74456 × 10^−12^ A	9.36139 mA/cm^2^	1.58058 × 1028 1/m^3^/s	0.64	62.93	3.76
Triple core NSs above PEDOT:PSS	4.00324 × 10^−11^ A	14.0162 mA/cm^2^	5.06522 × 1029 1/m^3^/s	0.98	73.0	10.02
Triple core NSs above P3HT:PCBM	3.60084 × 10^−12^ A	9.0021 mA/cm^2^	1.53888 × 1028 1/m^3^/s	0.62	62.08	3.46

## Data Availability

Not applicable.

## References

[1] Gan Q., Bartoli F.J., Kafafi Z.H. (2013). Organic Photovoltaics: Plasmonic-Enhanced Organic Photovoltaics: Breaking the 10% Efficiency Barrier. Adv. Mater..

[2] N’konou K., Torchio P. (2017). Optical absorption enhancement by inserting ZnO optical spacer in plasmonic organic solar cells. J. Nanophotonics.

[3] Masuko K., Shigematsu M., Hashiguchi T., Fujishima D., Kai M., Yoshimura N., Yamaguchi T., Ichihashi Y., Mishima T., Matsubara N. (2014). Achievement of More Than 25% Conversion Efficiency with Crystalline Silicon Heterojunction Solar Cell. IEEE J. Photovoltaics.

[4] Lv D., Jiang Q., Shang Y., Liu D. (2022). Highly efficient fiber-shaped organic solar cells toward wearable flexible electronics. npj Flex. Electron..

[5] Solak E.K., Irmak E. (2023). Advances in organic photovoltaic cells: A comprehensive review of materials, technologies, and performance. RSC Adv..

[6] Jahandar M., Kim S., Lim D.C. (2021). Indoor Organic Photovoltaics for Self-Sustaining IoT Devices: Progress, Challenges and Practicalization. Chemsuschem.

[7] Zisis C., Pechlivani E., Tsimikli S., Mekeridis E., Laskarakis A., Logothetidis S. (2019). Organic Photovoltaics on Greenhouse Rooftops: Effects on Plant Growth. Mater. Today: Proc..

[8] Jørgensen M., Norrman K., Gevorgyan S.A., Tromholt T., Andreasen B., Krebs F.C. (2012). Stability of Polymer Solar Cells. Adv. Mater..

[9] Zhang C., Zhou W., Sun S., Yi N., Song Q., Xiao S. (2015). Absorption enhancement in thin-film organic solar cells through electric and magnetic resonances in optical metamaterial. Opt. Mater. Express.

[10] Zeng B., Gan Q., Kafafi Z.H., Bartoli F.J. (2013). Polymeric photovoltaics with various metallic plasmonic nanostructures. J. Appl. Phys..

[11] Aneesh P., Kumar C.R., Varma P.R., Vivek K., Namboothiry M.A. (2015). Enhancement in Photovoltaic Properties of Plasmonic Nanostructures Incorporated Organic Solar Cells Processed in Air Using P3HT:PCBM as a Model Active Layer. Org. Photon Photovoltaics.

[12] Raman A., Yu Z., Fan S. (2011). Dielectric nanostructures for broadband light trapping in organic solar cells. Opt. Express.

[13] Jiang W., Salvador M., Dunham S.T. (2013). Combined three-dimensional electromagnetic and device modeling of surface plas-mon-enhanced organic solar cells incorporating low aspect ratio silver nanoprisms. Appl. Phys. Lett..

[14] Zheng D., Huang W., Fan P., Zheng Y., Huang J., Yu J. (2017). Preparation of Reduced Graphene Oxide:ZnO Hybrid Cathode Interlayer Using In Situ Thermal Reduction/Annealing for Interconnecting Nanostructure and Its Effect on Organic Solar Cell. ACS Appl. Mater. Interfaces.

[15] Tasnim S., Kaysir R., Islam J. Effect of Plasmonic Silver Nanoparticles Layer on The Performance of Organic Photovoltaic Cell. Proceedings of the 2021 International Conference on Electronics, Communications and Information Technology (ICECIT).

[16] Abhijith T., Suthar R., Karak S. Synthesis of Au-Ws2 Hybrid Nanostructures for Performance Enhancement in Organic Solar Cells. Proceedings of the 2022 IEEE International Conference on Emerging Electronics (ICEE).

[17] Duche D., Torchio P., Escoubas L., Monestier F., Simon J.-J., Flory F., Mathian G. (2009). Improving light absorption in organic solar cells by plasmonic contribution. Sol. Energy Mater. Sol. Cells.

[18] N’Konou K., Many V., Ruiz C.M., Treguer-Delapierre M., Torchio P. (2018). Effect of shell thickness of gold-silica core-shell nanospheres embedded in an organic buffer matrix for plasmonic solar cells. J. Appl. Phys..

[19] Ko D.-H., Tumbleston J.R., Zhang L., Williams S., DeSimone J.M., Lopez R., Samulski E.T. (2009). Photonic Crystal Geometry for Organic Solar Cells. Nano Lett..

[20] Duché D., Masclaux C., Le Rouzo J., Gourgon C. (2015). Photonic crystals for improving light absorption in organic solar cells. J. Appl. Phys..

[21] Li Q., Yoon W.J., Ju H. (2014). Optimization of an organic photovoltaic device via modulation of thickness of photoactive and optical spacer layers. Nanoscale Res. Lett..

[22] Lee J.K., Coates N.E., Cho S., Cho N.S., Moses D., Bazan G.C., Lee K., Heeger A.J. (2008). Efficacy of TiOx optical spacer in bulk-heterojunction solar cells processed with 1,8-octanedithiol. Appl. Phys. Lett..

[23] Li Y.-F., Kou Z.-L., Feng J., Sun H.-B. (2020). Plasmon-enhanced organic and perovskite solar cells with metal nanoparticles. Nanophotonics.

[24] Beck F.J., Mokkapati S., Polman A., Catchpole K.R. (2010). Asymmetry in photocurrent enhancement by plasmonic nanoparticle arrays located on the front or on the rear of solar cells. Appl. Phys. Lett..

[25] Shao P., Chen X., Guo X., Zhang W., Chang F., Liu Q., Chen Q., Li J., Li Y., He D. (2017). Facile embedding of SiO2 nanoparticles in organic solar cells for performance improvement. Org. Electron..

[26] N’konou K., Peres L., Torchio P. (2018). Optical Absorption Modeling of Plasmonic Organic Solar Cells Embedding Silica-Coated Silver Nanospheres. Plasmonics.

[27] Omrani M.K., Fallah H. (2018). Improving light trapping of polymer solar cell via doping a new array of triple core-shell spherical nanoparticles utilizing realistic modeling. Sol. Energy.

[28] Zhang R., Zhou Y., Peng L., Li X., Chen S., Feng X., Guan Y., Huang W. (2016). Influence of SiO2 shell thickness on power conversion efficiency in plasmonic polymer solar cells with Au nanorod@SiO2 core-shell structures. Sci. Rep..

[29] Erwin W.R., Zarick H.F., Talbert E.M., Bardhan R. (2016). Light trapping in mesoporous solar cells with plasmonic nanostructures. Energy Environ. Sci..

[30] Dang X., Qi J., Klug M.T., Chen P.-Y., Yun D.S., Fang N.X., Hammond P.T., Belcher A.M. (2013). Tunable Localized Surface Plasmon-Enabled Broadband Light-Harvesting Enhancement for High-Efficiency Panchromatic Dye-Sensitized Solar Cells. Nano Lett..

[31] Shabat M.M., Nassar S.A., Schaadt D.M. (2020). Simulation of three types of nanoparticles on solar cell structure model. Int. J. Mod. Phys. B.

[32] Pettersson L.A.A., Roman L.S., Inganäs O. (1999). Modeling photocurrent action spectra of photovoltaic devices based on organic thin films. J. Appl. Phys..

[33] Kadem B., Hassan A., Cranton W. Performance Optimization of P3HT:PCBM Solar Cells by Controlling Active Layer Thickness. Proceedings of the 31st European photovoltaic solar energy conference and exhibition, EUPVSEC.

[34] Yu H., Li Y., Dong Y., Huang X. (2016). Fabrication and Optimization of Polymer Solar Cells Based on P3HT:PC_70_BM System. Int. J. Photoenergy.

[35] Wibowo A., Marsudi M.A., Amal M.I., Ananda M.B., Stephanie R., Ardy H., Diguna L.J. (2020). ZnO nanostructured materials for emerging solar cell applications. RSC Adv..

[36] Drude P. (1900). Zur Elektronentheorie der Metalle. Ann. der Phys..

[37] Huang Q., Hu X., Fu Z., Lu Y. (2017). Plasmonic Thin Film Solar Cells. Nanostructured Solar Cells.

[38] Koul S., Hakim N.-U. (2018). Recent Advances in the Determination of Optimal Active Layer Thickness for Bulk Heterojunction Organic Solar Cells. Trans. Electr. Electron. Mater..

[39] Mahani F.F., Mokhtari A. Enhancement of ITO-free organic solar cells utilizing plasmonic nanohole electrodes. Proceedings of the 7th International Conference on Nanotechnology (ICN).

[40] Hasan M., Rahman S.A., Prodhan M.H., Talukder A.H. (2018). Absorption Enhancement of Organic Solar Cell using Aluminum Oxide as a Photonic Crystal. J. Bangladesh Acad. Sci..

[41] Yadav A.S., Tran D.T., Teo A.J.T., Dai Y., Galogahi F.M., Ooi C.H., Nguyen N.-T. (2023). Core–Shell Particles: From Fabrication Methods to Diverse Manipulation Techniques. Micromachines.

